# A Review of the Lateral Patellofemoral Joint: Anatomy, Biomechanics, and Surgical Procedures

**DOI:** 10.5435/JAAOSGlobal-D-21-00255

**Published:** 2022-07-20

**Authors:** Breana Siljander, Marc Tompkins, Juan Pablo Martinez-Cano

**Affiliations:** From the Department of Orthopedic Surgery, University of Minnesota, Minneapolis, MN (Dr. Siljander and Dr. Tompkins); the Tria Orthopedic Center, Bloomington, MN (Dr. Tompkins); the Gillette Children's Specialty Healthcare, St. Paul, MN, (Dr. Tompkins); the Fundación Valle del Lili, Departamento de Ortopedia, Colombia (Dr. Martinez-Cano); and the Universidad Icesi, Cali, Colombia (Dr. Martinez-Cano).

## Abstract

The lateral patellofemoral joint soft tissues contain key structures that surround and balance the joint. These structures can affect joint tracking, stability, and force distribution. It is important to understand the lateral patellofemoral anatomy and biomechanics, and their relationship with patellofemoral instability, anterior knee pain, and osteoarthritis. Lateral-sided surgical procedures such as lateral release, lateral retinacular lengthening, and partial lateral patellar facetectomy can be useful in the treatment of such patellofemoral problems.

The lateral patellofemoral joint (LPFJ) includes those soft-tissue structures that are laterally based, between the patella and the femur. These soft tissues include the lateral patellofemoral ligament (LPFL), the lateral patellomeniscal ligament (LPML), the lateral patellotibial ligament (LPTL), the vastus lateralis obliquus, the quadriceps aponeurosis, and the iliotibial band. Since retinaculum is defined as soft-tissue structures that stabilize a tendon, we will refer to these lateral structures collectively as the lateral retinaculum because they stabilize the extensor mechanism tendons. The lateral retinaculum structures are critical in the movement and stability of the patellofemoral joint. When there is laxity of these structures, usually iatrogenic, the patient can develop medial dislocation or instability.^1^ Meanwhile, when there is lateral tightness, often producing increased tilt, the patient can develop overload, anterior knee pain, and eventually lateral patellofemoral osteoarthritis (OA). In addition, undue tension in the lateral retinaculum can worsen the symptoms of lateral patellar instability.^2^

These patellofemoral problems can be addressed with lateral-sided surgical procedures. Lateral release and lengthening can be useful in cases of lateral patellar overload and lateral patellar OA and in certain cases of patellofemoral instability where lateral tightness is part of the underlying condition.^2^ Lateral patellar facetectomy can be used as a nonarthroplasty alternative surgery for isolated patellar OA of the lateral facet.^3^

The anatomy of the LPFJ is less well described than medial patellofemoral joint soft-tissue structures, and a summary of lateral-sided operations has not previously been published. This review outlines the LPFJ anatomy and biomechanics, as well as techniques and clinical outcomes of lateral-sided surgical techniques.

## Anatomy of Lateral Patellofemoral Joint

The LPFJ is the lateral aspect of the articulation between the femur and the patella. The stability of this joint is derived from the osseous congruity, static soft-tissue attachments, and dynamic muscular stabilizers. These lateral-sided structures balance the medial-sided forces of the medial patellofemoral structures to ensure congruent tracking of the patella through its range of motion. There is a lack of clarity in the literature on how many layers are present in the lateral structures, and these structures have been referred to by multiple names including the LPFL, deep transverse retinaculum, and epicondylopatellar band.^4^ For functional purposes in this review, we will approach these structures as having two layers (superficial and deep) and the joint capsule/synovium.

### Superficial Retinacular Layer

The superficial lateral retinacular layer is formed by the merge of arciform fibers from the iliotibial band, the quadriceps aponeurosis, and the vastus lateralis obliquus tendon.

#### Iliotibial Band

**Figure 1:**
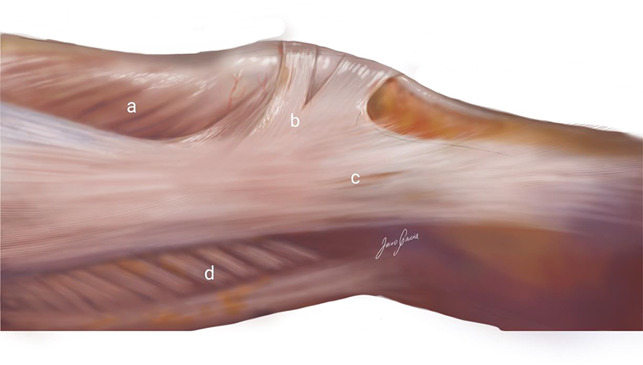
Illustration showing the arciform fibers and the insertions of the iliotibial band to the patella and Gerdy tubercle: (a) vastus lateralis muscle, (b) superficial oblique retinaculum (arciform fibers), (c) superficial layer of the iliotibial band, and (d) biceps femoris muscle.

As it travels distally, the iliotibial band has direct fibers that insert on Gerdy tubercle, but there are also arciform fibers of the iliotibial band that connect to the patella and patellar tendon. These are the most superficial fibers, and they lie superficial to or are contiguous with the anterior patellar periosteum (Figure 1).^5^

#### Vastus Lateralis Obliquus/Quadriceps Aponeurosis

Deep to the arciform fibers of the iliotibial band lies the tendon of the vastus lateralis obliquus and the quadriceps aponeurosis which attach variably to the lateral patella, but primarily on the proximal and superficial portions of the lateral patella. There is interdigitation between the iliotibial band and these structures.^6^

### Deep Retinacular Layer

**Figure 2:**
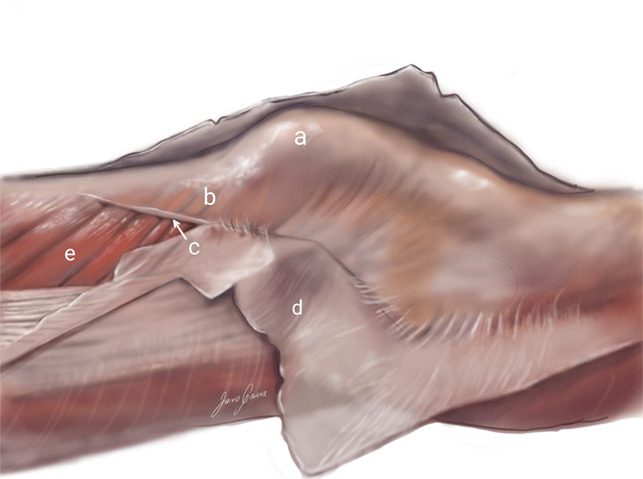
Lateral view of the knee with the superficial layer reflected showing (a) the patella, (b) the quadriceps aponeurosis, (c) the anchor of (d) the deep fascia to que quadriceps aponeurosis, and (e) the vastus lateralis.

The deep layer is formed by quadriceps aponeurosis and the deep fibers of the iliotibial band with insertions at the linea aspera (lateral intermuscular septum), lateral epicondyle, and Gerdy tubercle. Figure 2 shows this deep layer.^5,6^ The deep layer also contains the lateral patellofemoral, lateral patellomeniscal, and lateral patellotibial ligaments described below.

#### Lateral Patellofemoral Ligament

The LPFL is a thickening in the deep fascial layer that attaches to roughly the proximal half of the lateral patella and inserts, on average, just distal and anterior to the lateral femoral epicondyle.^6,7^

#### Lateral Patellomeniscal Ligament

The LPML is a thickening in the deep fascial layer just distal to the LPFL on the patella and travels from the lateral patella to the anterolateral aspect of the lateral meniscus.^6,8^

#### Lateral Patellotibial Ligament

**Figure 3:**
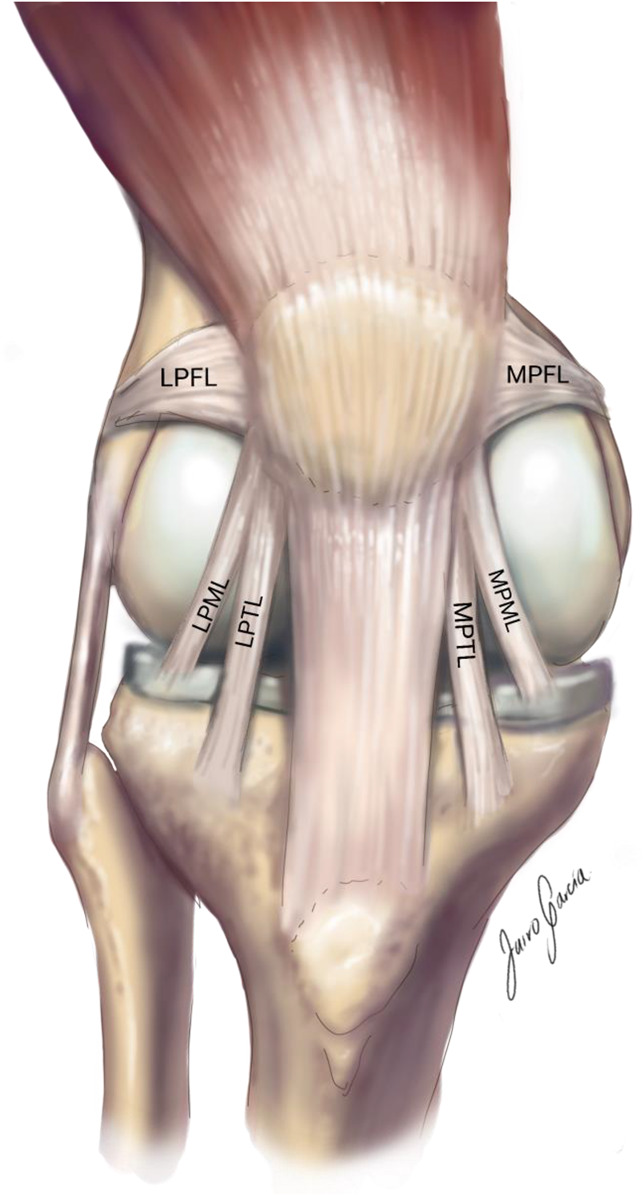
Illustration showing the lateral and medial patellofemoral ligaments (LPFL and MPFL), the lateral and medial patellomeniscal ligaments (LPML and MPML), and the lateral and medial patellotibial ligaments (LPTL and MPTL) [This drawing represents the authors' and an artist's rendering of the anatomy, and the capsule has been removed to simplify the visualization of these structures, but they are not intra-articular. In dissections, there is confluence of these ligaments with the capsule, they blend with the capsule but are not intracapsular].

The LPTL is a thickening in the deep fascial layer just distal to the LPML on the patella and travels from the lateral patella to the tibia inserting on Gerdy tubercle or between Gerdy and tibial tubercles.^4,6,9^ Figure 3 shows the patellofemoral joint ligaments.

### Joint Capsule/Synovium

**Figure 4:**
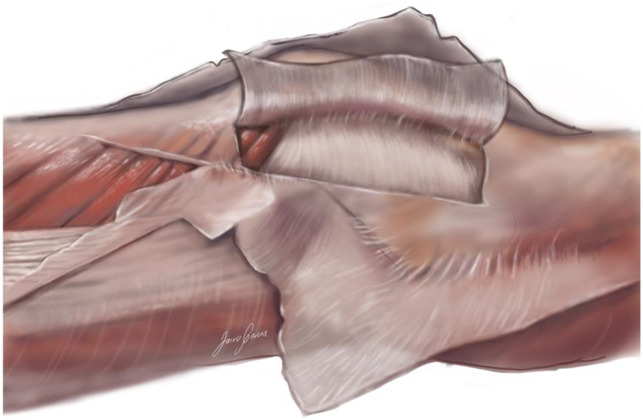
Illustration showing the lateral joint capsule in the back, the deep retinacular layer reflected up, and the superficial retinacular layer reflected down.

Older cadaveric studies suggested that the retinacular tissues were a thickening of the joint capsule, but more recent studies have reproducibly demonstrated that the deep retinacular layer can be separated from the joint capsule.^7-9^ Figure 3 shows the deep retinacular layer and Figure 4 the lateral joint capsule.

The superior and inferior lateral genicular arteries traverse the lateral retinaculum and can be encountered when performing lateral soft-tissue procedures. The superior lateral genicular artery branches from the popliteus artery and proceeds anteriorly at roughly the level of the metaphyseal flare; it stays just deep to the quad aponeurosis.^6,10^ The inferior lateral genicular artery branches from the popliteus artery and proceeds anteriorly at roughly the level of the joint line and also stays in the layer of deep retinacular tissues.^10^

### Biomechanics of Lateral Patellofemoral Joint

#### Superficial Retinacular Layer

##### Iliotibial Band

A tight IT band has been associated with lateral patellar tracking, which can exacerbate lateral instability.^11^ The arciform fibers running from the iliotibial band to the patella are perpendicular or oblique to both the iliotibial band and the patella. This also means that this connection is perpendicular or oblique to the direct line of longitudinal force for both the iliotibial band and the extensor mechanism helping to keep the patella from translating medially.^5^ The tensile strength of these fibers is 582 N, and the stiffness is 97 N/mm.^8^

### Vastus Lateralis Obliquus/Quadriceps Aponeurosis

During knee range of motion, the patella is guided to engage with the trochlea because of the posterior pull of the VMO and VLO on either side of the knee.^11,12^ The VLO force is directed 35° proximal-laterally off the femoral axis and counteracts the proximal-medial force of the VMO.^13^ An imbalance of these muscles can lead to differential stresses on the patellar facets.

### Deep Retinacular Layer

#### Lateral Patellofemoral Ligament

The size and shape of the LPFL have been studied in several studies suggesting some variability. It has been found to be a conoid ligament with one study demonstrating a patellar attachment width of 14.3 mm and a femoral attachment width of 11.7 mm.^7^ In another study, the length was found to be an average of 23.2 mm. The tensile strength of the LPFL has been found to be 172 N, and the stiffness is 16 N/mm.^8^

#### Lateral Patellomeniscal Ligament

Less is known about the dimensions of the LPML, but the tensile strength of the LPML has been found to be 85 N, and the stiffness is 13 N/mm.^8^

#### Lateral Patellotibial Ligament

The literature is lacking on the dimensions and strength of the LPTL.

### Joint Capsule/Synovium

The joint capsule can also become tight, which can contribute to LPFJ pathology.

### Combined Effect

The clinical significance of an insufficient medial patellofemoral ligament or complex is broadly recognized. The clinical implication of lateral retinacular insufficiency is less clear, but these tissues have been described as the primary stabilizers against medial patellar instability.^14^ Load sharing of the knee extensor mechanism demonstrated crucial loadbearing by the retinaculum that would otherwise be distributed to the quadriceps or patellar tendon.^15^ Cadaveric studies have demonstrated medial patellar subluxation after resection of the lateral retinaculum, with the superficial layer providing the most medial restraint at 30 degrees of knee flexion and the deep layer providing the most restraint in extension.^16^

## Lateral-sided Surgical Procedures for the Patellofemoral Joint

### Lateral Retinacular Release

#### Background

Lateral retinacular release (LRR) was first described by Pollard in 1891 and more recently in the 1970s by Willner. It was initially used as an isolated treatment for recurrent patellar dislocation, but soon was expanded to different patellofemoral problems, including pain and OA. Over time, it has been shown to have unpredictable results and variability in patient satisfaction, and therefore, it has lost popularity and has become more rarely performed as an isolated surgery.^17,18^

### Indications

Anterior knee pain is a major cause of consultation for problems around the knee.^19^ Isolated LRR is indicated in anterior knee pain when the pain is associated with excessive load in the LPFJ or tight lateral parapatellar soft-tissue structures; ideally, there should be no other issues such as chondral injury or dysmorphic bony morphology.^20^ The use of isolated LRR in patellar instability is controversial and not currently recommended.

### Surgical Technique

During this surgery, any of the lateral patellofemoral soft tissues which are tight are longitudinally incised, from the distal border of the vastus lateralis tendon to the tibial tubercle. The layers involved include the capsule as well as deep and superficial layers of the lateral retinaculum; which layers are included depend on whether the release is done arthroscopically or open. Arthroscopically, the capsule is incised with an arthroscopic cautery or ablator, and the release is extended superficially. If the release is extended through the superficial lateral retinacular layer, there can be protrusion of any effusion in this area. LRR performed through an open approach involves the superficial and deep retinacular layers but can sometimes spare the capsular layer. The release is considered sufficient when the patella can be tilted to neutral. If the geniculate arteries are encountered during the release, the surgeon should take care to cauterize them.

### Clinical Outcomes

#### Patellofemoral Instability

Lateral retinacular release was once used in patellofemoral instability as an isolated procedure; however, patient satisfaction after isolated lateral retinacular release for patellofemoral instability without any other surgery is variable, with satisfactory results ranging between 44% and 93%.^18,21^ Aglietti et al.^18^ found poor results when isolated LRR was conducted in patients with instability, and the results were inferior when compared with medial or distal realignment. Over time, it has been further shown that performing isolated LRR is not effective for patients with patellofemoral instability; good results do not persist over time, and recurrence is high.^18,22,23^ Patellofemoral instability is a multifactorial problem with multiple surgeries and combinations of surgeries used in the treatment, so LRR alone is insufficient to treat this condition.

#### Anterior Knee Pain

Isolated LRR can have good results when the pain is associated with excessive load in the LPFJ or tight lateral parapatellar soft-tissue structures.^20^ Aderinto et al.^24^ found improvement of symptoms in 80% of 50 patients with patellofemoral arthritis after isolated LRR, but 41% of the patients remained unsatisfied with their knee. Alemdaroglu et al.^25^ found notable improvement in pain and function with LRR plus cartilage débridement, after 2 years, in patients with patellofemoral arthritis with no instability or severe malalignment. The effectiveness of isolated LRR decreases when patellofemoral pain is associated with chondral injury (common in the patella), hyperlaxity, instability, or malalignment.^1,22,26^ Many patients with patellofemoral OA have association with trochlear dysplasia or malalignment.^26^ Outcomes for isolated LRR are worse in OA when these bony pathologies are present.^20,23^

#### Pitfalls

Potential complications of lateral release include hemarthrosis or excessive bleeding, medial instability, and burning the skin during the arthroscopic release.^27^ It is recommended to carefully cauterize bleeding vessels during the procedure, avoid excessive release, and not penetrate into the skin and subcutaneous tissue.

## Lateral Retinacular Lengthening

### Background

Lateral retinacular lengthening (LRL) was first described in 1973 by Slocum et al.^2^ It is an alternative to lateral release that offers a more controlled and balanced elongation of lateral-sided soft tissues when there is notable tilt and tightness.

### Indications

#### Anterior Knee Pain

Isolated LRL is indicated for patients with symptomatic tight lateral retinaculum without patellar instability, similar to those patients for LRR.^28^

#### Patellofemoral Instability

LRL is not done in isolation for patients with patellofemoral instability but is an additional tool in the surgical treatment for these patients. It is commonly paired with medial patellofemoral ligament reconstruction to improve the balance between medial and lateral translation of the patella when there is excessive lateral retinacular tension and patellar tilt.^19,29^ It is included in a recent algorithm for the management of recurrent lateral patellar dislocation when lateral patellar tilt is greater than 20°.^30^ It can be included with other concomitant procedures as well, such as tibial tubercle translation.^29^ Habitual dislocation in flexion is an infrequent entity but requires lateral release or lengthening to be able to center the patella over the trochlear groove; however, it is important to again emphasize that this is only one of the procedures needed in these complex cases.^30^

### Surgical Technique

**Figure 5:**
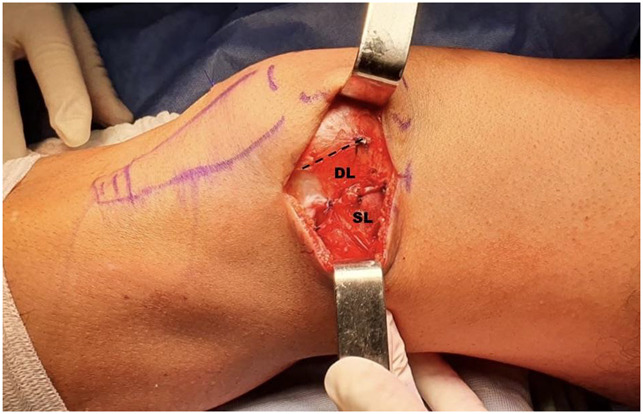
Photograph showing the surgical approach, lateral to the patella. The incision shows the superficial layer (SL) and the deep layer (DL) of the lateral retinaculum, sutured, and lengthened 15 mm. The dotted line shows the original attachment of the superficial layer.

**Figure 6:**
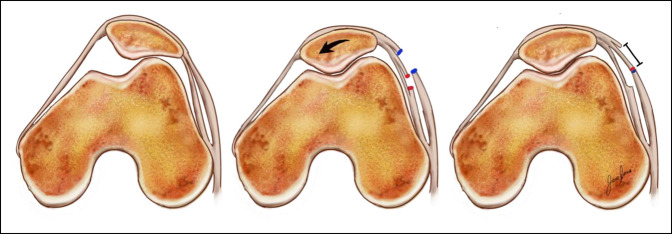
Illustrations showing the axial views of the patellofemoral joint and the effect of lateral retinacular lengthening. On the left, the original patellofemoral joint shows lateral tilt of the patella. On the center, the superficial (blue) and deep (red) layers of the lateral retinaculum have been incised with the correction of the lateral tilt. On the right, the superficial and deep layers are sutured lengthening the lateral retinaculum.

The open technique is done through a 3-cm longitudinal incision, 1 cm lateral to the patella. The superficial layer of the lateral retinaculum is incised first near the patella, and this is freed from the deep layer moving posterolaterally. Then, the deep layer of the lateral retinaculum is incised, protecting the lateral capsule. The incision extends proximally and distally similarly to the LRR, from the inferior border of the vastus lateralis tendon to the tibial tubercle. Finally, the free edges of the superficial and deep lateral retinaculum are sutured together (Figures 5 and 6). The aim is to have no tilt while maintaining medial patellar glide between 1 and 2 quadrants.

### Clinical Outcomes

Lateral release and lateral retinacular lengthening have been directly compared in a clinical trial from O'neill, which included 86 patients with anterior knee pain and lateral patellar tilting on Merchant radiographs. They were randomized to open lateral retinacular lengthening or arthroscopic release. Patients in the LRL group had markedly better clinical outcomes (Tegner and Lysholm score) compared with the LRR group. No notable difference was observed in range of motion, atrophy of the thigh, or perioperative complications.^31^

Pagenstert et al. compared open techniques for lateral release and lateral retinacular lengthening in a clinical trial with 28 patients and 2 years of follow-up. The study was limited to patients with retinacular pain, tight retinaculum, and decreased patellar mobility. The Kujala score was markedly better for the lengthening; the release was found to have greater quadriceps atrophy; and there were 5 cases of medial patellar subluxation with the release compared with no cases in the lengthening group.^32^

Some authors have suggested that medial subluxation after lateral release could be prevented with a more conservative release that has a goal of 1 to 2 patellar quadrants of medial patellar glide only, avoiding the over release of the retinaculum caused when using the 90° tilt-up end point.^28^ It may be easier and more reproducible, however, to lengthen instead of releasing the lateral retinaculum to control the final mobility of the patella.

Regarding arthroscopic release compared with open lengthening, the arthroscopic release is more cosmetic, but open lengthening has the advantage of offering better control of hemostasis, decreasing the risk of hemarthrosis.^33^ Lateral lengthening also more precisely balances medial to lateral patellofemoral forces, controlling the exact amount of tissue lengthening and decreasing the risk or medial patellar instability.

In summary, isolated LRL is indicated for treating lateral retinaculum tightness and anterior knee pain associated with lateral patellar overload. LRL is also useful as a coadjuvant in the treatment of patellofemoral instability with tight lateral retinaculum, but not as isolated treatment.^29^

### Pitfalls

Complications may include over release of the lateral retinaculum that could lead to medial instability. The risk of this complication, however, has been shown to be lower compared with lateral release.^32^ In addition, there is lower risk of bleeding or hemarthrosis because cauterization can be done to better control bleeding.

### Partial Lateral Patellar Facetectomy

#### Background

Semipatellectomy was first described by Sacks and further refined by O'Donoghue.^34^ During this surgery, a portion of the lateral facet of the patella (generally 10 to 15 mm) and any lateral osteophyte is resected. The net effect of patellar facetectomy is decompression of the LPFJ and functional lengthening of the lateral retinaculum.^3^ The lateral retinaculum contains a high density of nerve endings, and decompression is believed to relieve the source of notable pain.^35,36^ The desired outcome of this procedure is symptom relief rather and possible improvement of PFJ mechanics.^37^

#### Indications

Partial lateral patellar facetectomy is indicated in young, active patients with isolated symptomatic lateral patellofemoral osteoarthritis with lateral patellar tilt and osteophytes on the lateral border of the patellofemoral joint, who failed nonsurgical management.^38,39^ Ideally, the remainder of the cartilage of patellar facets is intact,^34^ and patellar tracking is normal (tibial tubercle-trochlear groove distance <20 mm) [Sanchis alfonso]. Patellar facetectomy is nearly always done with lateral retinacular release or lengthening.^38^ Indications for partial lateral patellar facetectomy in patients with trochlear dysmorphism or patellar instability are less clear.^28,34^

### Surgical Technique

#### Open Technique

**Figure 7:**
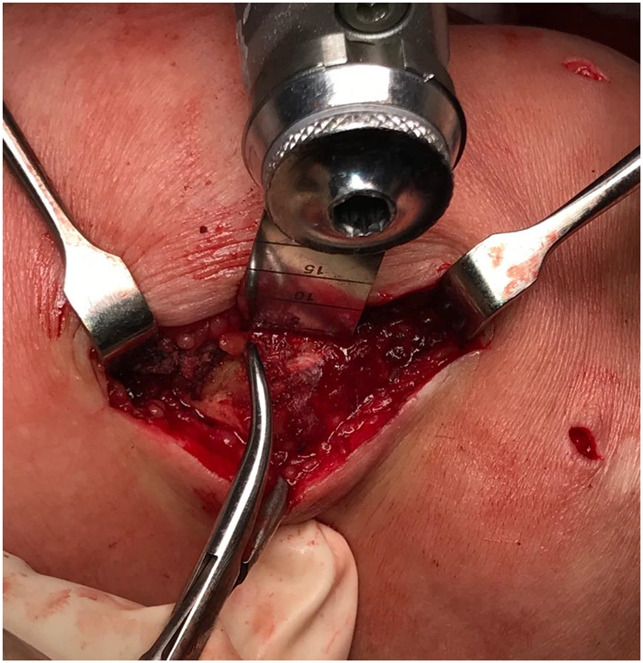
Photograph showing how the lateral facetectomy is done with an oscillating saw, while the lateral facet is held with a clamp.

**Figure 8:**
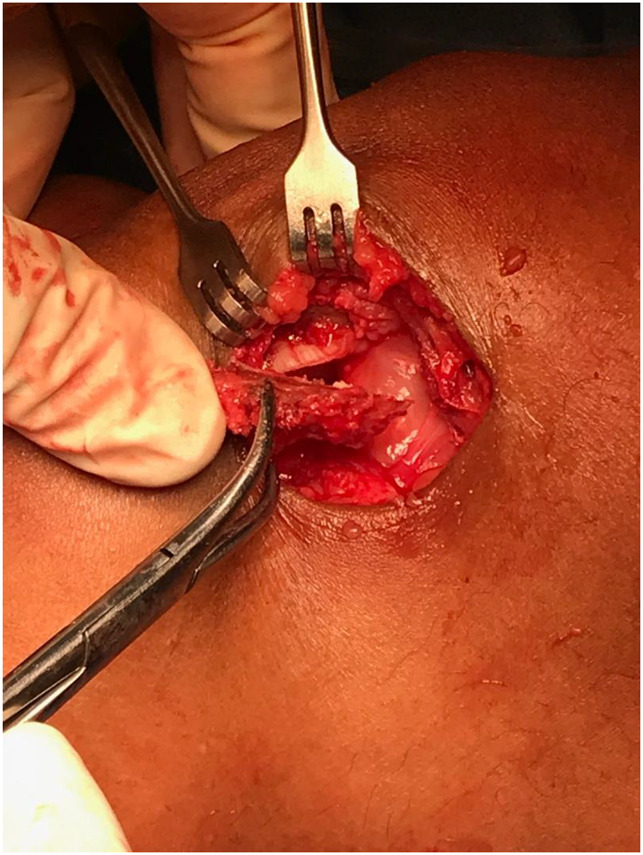
Photograph showing the final result after the lateral facetectomy has been done and the fragment is separated from the rest of the patella.

A longitudinal incision is made along the lateral patellar border, similar to that for an open LRL,^34^ and an LRL approach can be used to access the lateral capsule and lateral patellar facet. The soft tissues are then sharply dissected off the lateral patellar facet, and patellofemoral congruency is assessed.^37^ Patellar osteophytes are then excised. As shown in Figures 7 and 8, an oscillating saw, chisel, or rongeur is then used to resect 10 to 15 mm of the lateral patellar facet in a vertical or oblique osteotomy.^34,37^ Care is taken to avoid resecting >50% of the facet. The articular surface of the patella is assessed to ensure the erosions are removed. Cut bony surfaces are covered with bone wax.^37^ Patellar tracking is then assessed. Additional lateral retinacular release or lengthening can be done based on clinical need. The joint is then closed by suturing the synovium and lateral retinaculum to the periosteal fibers of the extensor mechanism on the lateral margin of the patella or in a similar manner to the layered closure of the LRL.^38^

#### Arthroscopic Technique

A diagnostic arthroscopy of the knee joint is done. The knee is then ranged to find the area of the facet with the most impingement against the trochlea.^39^ A radiofrequency probe is used to mark the diseased facet. The knee is brought into 20 degrees of flexion, and a 5.5 mm burr is used to resect the lateral osteophytes and the most prominent aspect of the lateral facet. Typically, the width of the burr is used to measure the extent of the resection. The knee is again ranged under arthroscopic visualization to confirm that the area of impingement has been addressed.

#### Clinical Outcomes

A preponderance of level III and IV studies suggests reassuring outcomes for patients who are well indicated for and undergo partial lateral patellar facetectomy. Yercan et al^37^ demonstrated maintenance of long-term functional improvement after partial lateral patellectomy, as measured by knee society scores. Lopez-Franco et al^40^ in a retrospective study on middle-aged to elderly patients who underwent lateral facetectomy for lateral patellofemoral arthritis with the 10-year follow-up found improved knee society scores in 84% of patients and 30% conversion rate to total knee arthroplasty. A prospective study of 20 patients with a mean follow-up of 2 years found lateral facetectomy with lateral retinacular release resulted in a “moderate-to-good” result in 90%, which is defined as pain improvement in all patients with some improvement in long distance walking and stair climbing.^38^ In a retrospective review of 155 patients who underwent patellar facetectomy and were followed for 10.9 years, survival rates at 5, 10, and 20 years were 85%, 67%, and 47%, respectively.^41^ Thirty-seven percent were revised to patellofemoral arthroplasty, total knee arthroplasty, or total patellectomy. Another retrospective study with 1 to 29 years of follow-up found excellent results in patients with patellar osteomalacia, good results in patients with trauma,^34^ and poor results in patients with degenerative disease. A small retrospective case review found satisfactory outcomes in patients with Outerbridge grade III and IV changes of the patella.^42^ Risk factors predictive of failure after patellar facetectomy include medial tibiofemoral pain, tibiofemoral OA, and limited extension.^43^ Interestingly, a combined procedure of lateral facetectomy, lateral retinacular release, and tibial tubercle medialization did not produce outcomes that were superior to isolated facetectomy alone.^1^ Despite the low-quality evidence on clinical outcomes after partial lateral patellar facetectomy, the available studies suggest that this low-risk procedure may preserve native knee anatomy and delay the need for more invasive surgical interventions, such as total knee arthroplasty. Although outside the scope of this paper's focus for an extensive discussion, it should be noted that good results can be achieved for lateral patellofemoral arthritis with an anteromedialization tibial tubercle osteotomy.

Failure of a partial lateral patellar facetectomy can be defined as the need for conversion to patellofemoral arthroplasty, total knee arthroplasty, or patellectomy.^41^ It has been demonstrated that patellar facetectomy can be converted to a total knee arthroplasty without jeopardizing outcomes.^37^ Therefore, the consequence of a failed lateral facetectomy is small when a reliable salvage option is available.

### Pitfalls

Underresecting the lateral patellar facet or leaving part of the lateral osteophytes can result in continued lateral overload and pain. Overresecting the lateral patellar facet can affect a future arthroplasty by altering the shape of the patella, the soft-tissue attachments, and stability of the patella. Delaying range of motion can alter the patella tracking and produce stiffness. It is therefore important to be precise about the amount and location of resection, as well as to get motion started immediately after surgery.^3,39^

## Summary

Lateral-sided surgical procedures are useful in the treatment of patellofemoral joint problems. These include lateral release, lateral retinacular lengthening, and partial lateral patellar facetectomy. Lateral retinacular lengthening has a role in patellofemoral instability balancing, as well as in anterior knee pain and OA when there is increased lateral pressure or lateral tilt. Partial lateral patellar facetectomy can be a nonarthroplasty alternative for patients with patellofemoral OA. Understanding the lateral patellofemoral anatomy and biomechanics is important to indicate and perform these procedures.
